# Micronutrient intake and nutritional status in 16-to-24-year-olds adhering to vegan, lacto-ovo-vegetarian, pescatarian or omnivorous diets in Sweden

**DOI:** 10.1007/s00394-025-03738-2

**Published:** 2025-06-26

**Authors:** Isabelle Mulkerrins, Anine Christine Medin, Synne Groufh-Jacobsen, Claire Margerison, Christel Larsson

**Affiliations:** 1 Department of Food and Nutrition, and Sport Science, Faculty of Education, University of Gothenburg, PO Box 300, Gothenburg, SE-405 30 Sweden; 2 Department of Nutrition and Public Health, Faculty of Health and Sport Sciences, University of Agder, Universitetsveien 25, Kristiansand, 4630 Norway; 3 Institute for Physical Activity and Nutrition, Deakin University, Geelong, Australia

**Keywords:** Dietary intake, Plant-based, Vitamin D, Vitamin B_12_, Iron, Iodine

## Abstract

**Purpose:**

The nutritional adequacy of plant-based diets for youth is of public health relevance but is insufficiently studied. This cross-sectional study aims to assess the micronutrient intake and nutritional status in youth adhering to vegan, lacto-ovo-vegetarian, pescatarian or omnivorous diets in Sweden.

**Methods:**

Healthy 16-to-24-year-olds (*n* = 60 vegans, *n* = 59 lacto-ovo-vegetarians, *n* = 55 pescatarians, *n* = 61 omnivores) completed a 255-item web-based questionnaire, conducted four non-consecutive 24-hour dietary recalls, provided capillary blood samples and one spot urine sample. Usual micronutrient intakes were estimated by the Multiple Source Method. At the group level, risk of inadequate intake was defined as > 50% with intakes below average requirements. Samples were analysed for haemoglobin (HB), serum transferrin receptor (sTfR), homocysteine (tHcy), methylmalonic acid (MMA), 25-hydroxyvitamin D3 (25-OH-D3), and urinary iodine concentration (UIC).

**Results:**

From foods and supplements, all dietary groups demonstrated a risk of inadequate intakes of vitamin D and selenium, additionally vitamin A and calcium in vegans, B_12_ in lacto-ovo-vegetarians, and potassium in pescatarians. Median blood marker values of HB, sTfR, tHcy and MMA were within normal ranges across dietary groups. Insufficient 25-OH-D3 concentration (< 50 nmol/L) was observed in vegans, lacto-ovo-vegetarians, and omnivores. A significantly higher proportion of vegans were at risk of vitamin D deficiency (25-OH-D3 < 30 nmol/L) than pescatarians and omnivores. Median UIC (< 100 µg/L) indicated a risk of inadequate iodine intake in vegans, pescatarians, and omnivores.

**Conclusion:**

Youth, regardless of dietary practice, need support to ensure adequate micronutrient intakes, particularly for vitamin D and selenium. Further research is required to evaluate iodine nutrition in Swedish youth.

**Supplementary Information:**

The online version contains supplementary material available at 10.1007/s00394-025-03738-2.

## Introduction

The World Health Organization (WHO) supports that well-planned plant-based diets including vegan and lacto-ovo-vegetarian diets, with appropriate supplementation, can be nutritionally adequate throughout the life-course [1, 2]. Yet, such diets without supplementation or fortified foods can pose a nutritional challenge, particularly for vegans, due to lower quantities and lower bioavailability of several essential nutrients including vitamin B_12_ (B_12_), vitamin D, calcium, iron, zinc, selenium, iodine and omega-3 fatty acids in unfortified plant-based foods compared to animal-sourced foods [3–5]. The potential to consume adequate intakes of micronutrients when adhering to a plant-based diet is impacted by country-specific policies such as fortification strategies, as well as cultural contexts including cultural food habits, food availability, and geographical location. For example, in most Nordic countries vitamin D is identified as a nutrient of concern among the general population due to intakes below recommendations and limited UVB exposure during winter [6–8]. Consequently, plant-based diets with limited food sources of vitamin D will increase the risk for nutritional inadequacy of vitamin D.

To address low intakes of vitamin D in the general population in Sweden, a mandatory vitamin D fortification policy was implemented, which was updated in 2018/2019 [9, 10]. The updated policy entailed an increased fortification of some cow´s milk and dairy yoghurt products, and also included organic versions and plant-based alternatives (providing 0.75–1.10 µg vitamin D per 100 g), additionally margarine (providing 19.5–21.0 µg vitamin D per 100 g) [10]. Milk and dairy products are commonly consumed in Sweden, and provide approximately 13% of adolescent’s daily energy intake [11]. Furthermore, in Sweden plant-based foods are allowed to be fortified, which differs to countries such as Norway where fortification of foods is largely limited [9, 12]. Notably, the majority of plant-based milks and yoghurt alternatives on the Swedish market are fortified with B_12_ (73%), calcium (73–79%), and B_2_ (59–66%). However, only 19% are fortified with iodine [13]. Furthermore, most plant-based meat analogs in Sweden are not fortified [14]. The Swedish Food Agency supports that well-planned plant-based diets, which are appropriately supplemented, can be healthy and nutritionally adequate [15, 16]. Although emphasis is placed on the importance of having basic nutrition knowledge if following a vegan or vegetarian (lacto-, ovo-, lacto-ovo-vegetarian) diet to ensure that animal-sourced foods are replaced with nutritionally similar plant-based foods. Additionally, it is specified that the vegan diet should be complimented with supplementation of B_12_ and vitamin D, or that sufficient intakes of fortified products are ensured [15]. The same dietary recommendation applies to the vegetarian diet if intakes of milk, dairy products, and eggs is limited [16].

Previous studies have identified iodine as a micronutrient of concern when adhering to a plant-based diet, in particular a vegan diet, since animal-sourced foods are the main dietary sources of iodine [12, 17]. However, in Sweden most table salts are fortified with iodine (50 µg/g) [18]. Iodized table salt is used in the majority (75%) of Swedish households [8, 19], and is a key dietary source of iodine. It is estimated that iodized household salt contributes to at least 50% of Swedish adults iodine intake [20], with saltwater fish, cow´s milk, dairy products and eggs being other important dietary sources [20, 21]. Adequate iodine intake has been found among healthy, non-pregnant, non-lactating adults in Sweden, as well as in children/adolescents [8, 18, 21]. To-date no studies have evaluated the iodine nutrition in youth adhering to plant-based diets in Sweden.

At present, one in ten young adults adhere to a vegan or lacto-ovo-vegetarian diet in Sweden [22]. For youth [23] who are in a stage of life with heightened nutritional demands of some essential micronutrients (e.g., calcium [6] and iron [6]) due to growth and physiological development it is important to examine if nutritional requirements are met when eating plant-based diets. To the best of our knowledge, only two studies have been conducted among youth in Nordic countries eating plant-based diets. Whereof one study from 2022 among Norwegian 16-to-24 year olds (*n* = 165) eating plant-based (vegan, lacto-ovo-vegetarian, pescatarian, flexitarian) or omnivorous diets [12], and one study from the late 1990´s on Swedish 16-to-20 year olds (*n* = 60) adhering to a vegan or omnivorous diet [24]. Thus, this study aims to assess the micronutrient intake and nutritional status in youth adhering to vegan, lacto-ovo-vegetarian, pescatarian or omnivorous diets in Sweden.

## Methods

## Study design and recruitment

This cross-sectional study is part of a larger research project named *VeggiSkills-Sweden.* The project aims to assess the nutritional consequences of consuming plant-based diets of differing strictness among youth in Sweden, and to investigate food literacy, food habits, and health behaviours in relation to their dietary practice. Furthermore, the research project is part of an internationally collaborative project, where a similar research project was conducted in Norway, i.e., *VeggiSkills-Norway* [12]. This present study was conducted one year after the study in Norway.

In this present study, between December 2022 and January 2024, a total of 244 healthy participants were recruited by convenience and snowball sampling in Gothenburg, Sweden and nearby municipalities. Participants visited a research facility at the University of Gothenburg to complete several measurements on site. During the visit, all participants filled in a 255-item web-based questionnaire, completed a 24-hour dietary recall (24HDR), provided non-fasted blood and urine samples, and had their anthropometrics measured. Following the visit, participants were asked to conduct three non-consecutive 24HDRs on their own. More details about the study design, recruitment, and methods have been published previously [25].

### Eligibility criteria

Eligibility criteria included, ´aged 16-to-24 years´, ´healthy with no chronic or acute illness´, ´having adhered to their current dietary practice (vegan, vegetarian, pescatarian, omnivorous) for a minimum of six months with no plans to alter their current dietary practice´, ´not be pregnant, lactating, or have children´, ´living in Gothenburg or nearby municipalities´, ´comprehend Swedish´, and ´agree to physically visit the research facility once for measurements´.

## Sample size calculation

The sample size aimed for in the research project was calculated a priori by using Coles equation [26]. In the equation we used standard deviations from nationally representative dietary intake data of Swedish 17–18 year olds (both sexes) [8]. To determine which differences were meaningful to detect in key selected micronutrients, we used results from a previous Swedish study on young vegans and omnivores [24]. At least 16 females and 40 males were required per dietary group, to detect a difference between two dietary groups (vegans and omnivores) for intakes of 2 µg vitamin D, 2 µg B_12_, and 2 mg iron with 80% power and a significance level of 0.05. To account for dropouts, and with the intent to stratify by sex, we aimed to recruit 60 participants per dietary group.

## Categorization into dietary groups and exclusion of participants

To categorize participants into dietary groups, participants were asked in the questionnaire to self-identify the dietary practice they had followed in the past six months (´vegan´, ´ovo-vegetarian´, ´lacto-vegetarian´, ´lacto-ovo-vegetarian´, ´pescatarian´, ´flexitarian´, ´omnivore´). A description of which animal-sourced foods were included/excluded in the dietary practice was provided in the response alternatives. Their self-identified dietary practice was then cross-checked with their responses to a question which assessed how often (´Never´, ´Rarely´, ´Sometimes´, ´Daily´) they had included different animal-sourced foods (´milk and/or dairy products, including in foods and/or cooking´, ´egg, including in foods and/or cooking´, ´fish/seafood, including fish products´, ´poultry and/or poultry products´, ´red meat and/or red meat products´) in their diet in the past six months. They were categorized as vegan if they selected ´never´ for inclusion of all the animal-sourced food groups [27]. They were categorized as lacto-ovo-vegetarian if they selected ´daily´, ´sometimes´ or ´rarely´ for inclusion of dairy products and/or eggs, but ´never´ for inclusion of fish/seafood or meat (any type) [27]. They were categorized as pescatarian if they selected ´daily´, ´sometimes´ or ´rarely´ for inclusion of fish/seafood and ´never´ for inclusion of meat (any type), regardless of inclusion of dairy products/eggs [27]. They were categorized as omnivore if they selected ´daily´, ´sometimes´ or ´rarely´ for inclusion of meat (any type) and at least one of the other animal-sourced food groups [27].

When categorizing into dietary groups, seven participants were excluded from the study due to dietary practice ineligibility. Six were excluded due to self-identifying adherence to a flexitarian diet and reporting a limited intake of animal-sourced food groups. The participants´ flexitarian dietary practice was confirmed by their responses in a dietary screener [25, 28] in the questionnaire which assessed consumption frequency of several different food groups in the past six months. The participants´ responses showed that they consumed meat (any type) once a week or less often [27]. Additionally, one participant self-identified following a pescatarian diet, but contradicted this by reporting inclusion of red meat in their diet. This was confirmed by the participant´s response in the dietary screener [25, 28] assessing food group consumption frequency. Their response showed monthly intake of meat in the past six months, and therefore this participant was excluded. Furthermore, upon examination of the 24HDRs, one participant categorized as lacto-ovo-vegetarian reported fish consumption once. However, in the dietary screener [25, 28] the participant´s responses showed no intake of fish/seafood in the past six months, and therefore no inconsistency with the self-identified dietary practice existed and thus, this participant was not recategorized or excluded.

## Sociodemographic, health and lifestyle variables

To assess education level, participants were asked in the questionnaire to report their highest completed educational level (´Less than 12 years´, ´12 years [high school degree]´, ´University, less than 3 years´, ´University, 3 years or longer´ or ´Other/not applicable´). To assess health and lifestyle habits, participants were asked to specify, in the past six months, how often they had used tobacco/nicotine products i.e., cigarette, e-cigarette, and snuff (´Never´, ´Rarely´, ´Sometimes´, ´Daily´), consumed alcoholic beverages (´Never´, ´1 times a week or less often ´, ´1 times/week´, ´2–3 times/week´, ´4 times/week or more often´, ´Everyday´), and did moderate intensity and vigorous intensity physical activity (´Never´, ´Less often than once a week´, ´1–2 times/week´, ´3–4 times/week´, ´5–6 times/week´ and ´6 + times/week´). In the questionnaire moderate intensity was described as ´activities that are physically demanding and make you breathe somewhat harder than normal, e.g., brisk walking, jogging, cycling, and gym workouts´. Vigorous intensity was described as ´[…] very demanding and make you breathe much harder than normal, such as running, swimming, cycling, aerobics´.

## Diet duration

To assess diet duration, participants were asked in the questionnaire to report how long they had adhered to their current dietary practice. The predefined responses were, ´Less than 6 months´, ´6–11 months´, ´1–3 years´, ´3–5 years´, ´5–9 years´, ´10 + years´ and ´Whole life´.

## Habitual supplementation, and use of iodized household salt

To assess habitual supplementation, participants were asked in the questionnaire to report how often they had consumed twelve different dietary supplements in the past six months. The predefined responses were, ´Never´, ´1–3 times/month´, ´1–2 times/week´, ´3–4 times/week´, ´5–6 times/week´, and ´Daily´. If other types of supplements were consumed, participants were asked to describe the supplement and consumption frequency in an open textbox. A habitual supplement user was categorized as having consumed a supplement at least once a week.

Due to the widespread use of iodized table salt in Sweden, participants were asked in the questionnaire to report the type of household salt they used regularly. Photos (*n* = 8) of the most common household salts were shown. Participants who reported use of iodized salt (any type) were categorized as consumers of iodized salt.

## Assessment of dietary intake, 24-hour dietary recalls (24HDRs)

Dietary intake data, including supplements, was obtained through repeated non-consecutive 24HDRs completed in a web-based dietary assessment program, Nutrition Data (Nutrition Data Sweden AB, Sweden). A maximum of two 24HDRs were from a weekend. Briefly, the first 24HDR was completed at the research facility as an interview following the multiple pass method. Concurrent with the interview, the interviewer (first author) entered the dietary intake into the web-based dietary assessment program. Participants were first asked to recall their entire intake the previous day. Then they were asked for a detailed reporting of their intake for each mealtime separately. Specific details about the foods consumed were obtained to be able to select the correct food items in the web-based program. Additionally, for each mealtime, probing questions were asked about cooking methods, condiments, and beverages. Participants were also asked to estimate the portion size consumed for each food. To do this, they were instructed to use a portion guide booklet originally developed by the Swedish Food Agency (published 2010, Uppsala, Sweden) consisting of portion size photos for 24 foods. Also, household measurements (dl, tbsp, tsp) were used. Details about how the original portion guide booklet was developed have been published previously [29], and also how the booklet was updated for this present study with additional portion size photos for seven foods [25]. Subsequently, participants were asked about the time and place for their food intake the previous day. Thereafter the dietary intake entered into the web-based program was reviewed in full, also probing for any additional foods consumed between the reported mealtimes. Finally, participants were asked about intakes of often forgotten foods (e.g., sweets and snack foods, beverages, condiments, added fats) and supplements.

Following the research facility visit, participants were asked to conduct three 24HDRs on their own in the web-based program. They were provided the portion guide booklet and written instructions on how to complete the 24HDR in the same way as the first 24HDR. Unannounced messages were sent via email and text message on different days indicating when they were to complete a 24HDR. For each participant, we aimed to capture dietary intake from different days of the week. A maximum of 3 reminders were sent per recall, and participants completed their 24HDRs within 2–7 weeks. Participants who only completed one out of the four 24HDRs were excluded from this study, since at least two days of dietary intake are required to estimate usual intakes.

### Assessment of micronutrient intake

The web-based program automatically calculated energy and micronutrient intake using the Swedish Food Composition Database (version 20230613, Swedish Food Agency). There was comprehensive nutritional information for approximately 2,300 food items, which was based primarily on laboratory analyses. The Swedish Food Composition Database contained nutritional values for different types of plant-based meat analogs and dairy alternatives (both fortified and unfortified) [30]. Furthermore, there were additional food items available in the web-based programs database. These food items had been nutritionally calculated by Nutrition Data, using the products ingredient list, and selecting foods from the Swedish Food Composition Database to match the products nutrition information. However, for items not available in the databases (e.g., specific supplements, new plant-based foods) participants were instructed to describe the item in an open textbox (type, brand, quantity) or select a nutritionally similar food item available in the program. All food items specified in the open text box were entered into the 24HDR. As a first step, we compared the nutritional information available for the specified food item with similar items available in the Swedish Food Composition Database. When doing this, we considered the main ingredient of the product and the potential fortification of the food (using the ingredient list) to match the item to a food available in the database, with regards to energy and macronutrients, and if possible, micronutrients. If no suitable match was found, the nutrition information available for the product from the company website was used directly. Thus, acknowledging that this lacked comprehensive micronutrient values. For certain premade composite foods such as a vegan/vegetarian hamburger from a fast-food restaurant, a standardized recipe was created. To do this, we used the ingredient list for the food item available on the fast-food restaurants website, and matched the recipe as best as possible to the nutritional content available for the food item on the website. While for food items which could be either homemade or storebought, for example vegan cinnamon buns and vegan chocolate cake, standardized recipes were created using a popular online Swedish recipe, and based on typical ingredients, including fortified products where applicable. This approach was taken to approximate the nutritional composition as realistically as possible in the absence of complete product-specific data. For supplements, we obtained the nutritional content for the specific supplement using nutritional information provided by the company website and was manually entered into the web-based program.

## Estimation of usual daily intakes and evaluation of risk of inadequacy based on dietary intake

Usual daily intakes of micronutrients from foods only were estimated by the statistical Multiple Source Method (MSM), a two-part regression model, which uses the 24HDR intake data and adjusts for intravariability in intakes. The method is described in detail elsewhere [31, 32]. Discretionary sources of iodized household salt reported in the 24HDRs were excluded.

For total intakes of micronutrients (food and supplements combined), the estimated daily intake of micronutrients from supplements (reported in the 24HDRs) was summed together with the usual micronutrient intake from foods only (from the MSM). To estimate the daily intake of micronutrients from supplements, the nutritional value of supplements which were reported in the 24HDRs was used in combination with the reported consumption frequency data from the questionnaire. For example, if the consumption frequency was reported as 3–4 times/week we calculated the daily intake from the supplement(s) as follows, [supplement*3.5 / 7]. Participants who reported a supplement in the 24HDRs but did not report consumption frequency in the questionnaire were excluded from these calculations.

Risk of micronutrient inadequacy was evaluated by comparing the individual usual intakes to the age- and sex appropriate average requirements (AR) by the Nordic Nutrition Recommendations 2023 (NNR2023) [6]. At the group level, risk of inadequacy was defined as > 50% having usual intakes below the AR [33].

## Collection of blood and spot urine samples

At the research facility visit, non-fasted dried blood spots (DBS) [34] and spot urine samples were collected at random times during the day (between 08:30 − 19:30). Capillary blood samples were collected on a filter paper (Whatman™ 903) which were subsequently left to dry in room temperature with no direct sunlight for at least 3 h. Thereafter the DBS samples were stored at -80ºC until transported to Vitas Analytical Services™, Norway, for analysis of serum Transferrin Receptor (sTfR), homocysteine (tHcy), methylmalonic acid (MMA), and serum 25-hydroxyvitamin D3 (25-OH-D3). A blood drop was also collected in a microcuvette, and haemoglobin (HB) value was analysed directly using a HemoCue HB 801^®^ (HemoCue^®^ AB, Ängelholm, Sweden).

The spot urine samples were collected in 100 ml urine beakers (Vacuette^®^, Greiner Bio-One, GmbH, Austria). A subsample of urine was withdrawn to 7 ml Vacuette^®^ urine test tubes and stored at -80ºC until transported to the Hormone Laboratory, Oslo University Hospital, Norway, for analysis of urinary iodine concentration (UIC).

### Biochemical analysis

For analysis of sTfR three DBS punches (for all punches, 3.2 mm circle) were diluted with 250 mL of the kit sample diluent and placed at 4ºC over night. Thereafter analyzed using the EIA-5925 sTfR Human ELISA kit (DRG Instruments Gmbh, Marburg, Germany). The within-and between assay repeatability was 4.6–10.4% and 11.6–13.3%, respectively. The lower limit of quantification (LLOQ) was 0.45 mg/L.

For analysis of tHcy and MMA, four DBS punches were diluted with 225 µL water and 25 µL propanol. A mix of stable isotope labelled amino acids was added and used as internal standards. Samples were derivatized using propyl chloroformate and extracted into isooctane before being analysed by GC-MS on an Agilent 6890 GC (Agilent Technologies, Palo Alto, USA). For tHcy there was a within- and between assay repeatability of 3% and 13%, respectively, and for MMA it was 8% (within-assay) and 22% (between-assay). The LLOQ was 0.1 µmol/L for tHcy and 0.04 µmol/L for MMA.

For analysis of serum 25-OH-D3, analyte(s) were extracted from four DBS punches, and protein precipitated using 180 µL 2-propanol containing a stable isotope labelled internal standard. Thereafter analysed in an Ultivo Triple Quadrupole LC/MS (Agilent Technologies, Santa Clara, USA). The within- and between assay repeatability was 4.1–6.5% and 5.3–10%, respectively. The LLOQ was 20 nmol/L. The laboratory complies with DEQAS for the standardized analysis of vitamin D.

For quantification of UIC, samples were analysed by the colorimetric method, Sandell-Kolthoff´s reaction. The method is based on the catalytic effect of iodine on the red/ox reaction between arsenic and cerium. Analysis was performed using Delfia microtitration plates (Wallac Oy, Danmark), and read on 405 nm Tecan plates from Triolab (Perkin Elmer, Finland). The coefficient of variation was 19% at concentrations of 0.48 µmol/L, 10% at 0.92 µmol/L, and 7% at 3.1 µmol/L. The LLOQ was 0.2 µmol/L.

Due to an insufficient volume of blood or analytical issues there were missing values for HB (*n* = 5), sTfR (*n* = 3), MMA (*n* = 45), tHcy (*n* = 5), and 25-OH-D3 (*n* = 16).

## Evaluation of nutritional deficiencies based on biomarkers

To evaluate iron deficiency, a HB value of < 120 g/L (females) and < 130 g/L (males) was categorized as a risk of anaemia [35]. sTfR was analysed as a biomarker of iron insufficiency [36]. At present, sTfR lacks internationally established cut-off values. Normal range of sTfR for healthy populations according to the assay and kit-manual was 0.48–3.09 mg/L. Elevated values indicate insufficient iron for erythropoietic activity [36].

To evaluate B_12_ deficiency, tHcy and MMA were assessed. Both tHcy and MMA elevate as a result of limited B_12_ for metabolic function [37, 38]. MMA is a sensitive and specific biomarker of B_12_ deficiency, while tHcy values can also elevate due to folate or B_6_ deficiency [37, 38]. Elevated tHcy was categorized as > 15 µmol/L [39]. For MMA, elevated values were categorized as > 0.27 µmol/L [39], whereof depletion between > 0.27–0.37 µmol/L, and risk of deficiency as > 0.37 µmol/L.

To evaluate vitamin D deficiency based on serum 25-OH-D3, cut-off values from the Institute of Medicine [6, 40] were applied. Insufficient concentration was categorized as < 50 nmol/L, whereof suboptimal concentration as 30–49 nmol/L, and risk of deficiency as < 30 nmol/L.

To assess iodine status, median UIC [41] was evaluated as recommended by WHO [42]. We used the cut-offs from 2007, applicable for populations aged ≥ 6 years, and non-pregnant or lactating adults [42]. At the group level, increased risk of iodine deficiency was categorized as median UIC < 100 µg/L, whereof mild deficiency as 50–99 µg/L, moderate deficiency as 20–49 µg/L, and severe deficiency as < 20 µg/L. Adequate iodine status was categorized as median UIC of 100–199 µg/L.

## Anthropometric measurements

At the research facility visit, the participants height and body weight were measured to the closest 1 mm and 0.1 kg, respectively, while they were standing, wearing light clothing, and with no shoes on. A wall-mounted stadiometer (Hyssna M, Sweden) and digital scale (Beurer GmbH, Germany) were used. BMI was calculated as kg/m^2^.

## Statistical analyses for tests of differences between dietary groups

For data processing and analysis, Microsoft Excel (Microsoft Excel 365^®^, USA) and SPSS Version 29 (Chicago, IL, USA) were used. To test for differences between dietary groups in categorical variables (e.g., sociodemographic, health and lifestyle variables, diet duration, use of supplements, use of iodized table salt, proportion not meeting the ARs, and proportion at risk of nutritional deficiencies) we used crosstabulations with Chi-Square or Fischer’s Exact test. Continuous variables were evaluated for normal distribution using Shapiro-Wilks test, and by visually assessing Q-Q plots and histograms. One-way ANOVA was used to test for differences between dietary groups in age and BMI, and results are presented as mean values with standard deviations (SD). The Kruskal-Wallis test with pairwise comparisons was used to test for differences between dietary groups in usual intakes of micronutrients and nutritional status, and results are presented as median values with 25th and 75th percentiles. To correct for multiple analyses Bonferroni post hoc test was employed. To test for differences in nutritional status between habitual vs. non-habitual supplement users within each dietary group, the Mann Whitney U test was performed **(supplementary material)**. Further, we investigated if there were differences in 25-OH-D3 concentrations depending on season of participation using Kruskal-Wallis and Mann Whitney U tests **(supplementary material)**. For all tests, adjusted p-value of < 0.05 (two-sided) was accepted as statistically significant.

## Results

## Participant characteristics

Participant characteristics and percentage of supplement users (habitual use and percentage reporting supplements in the 24HDRs) are presented in Table 1. A total sample of 244 youth were recruited to this study, however nine were excluded; seven due to dietary practice ineligibility and two due to only completing one out of the four 24HDRs. Thus, the final study sample consisted of 235 youth aged 16–24 years, whereof 60 vegans, 59 lacto-ovo-vegetarians, 55 pescatarians, and 61 omnivores. Across dietary groups the majority were female (78% in the total sample), and therefore this study is underpowered to detect differences, stratified by sex, between the dietary groups as described in the methods section.

**Table 1:** Participant characteristics and percentage of supplement users among youth in Sweden adhering to vegan, lacto-ovo-vegetarian, pescatarian or omnivorous diets (n = 235)

	Total sample (n = 235)		Vegan (n = 60)		Lacto-ovo -vegetarian (n = 59)		Pescatarian (n = 55)		Omnivore (n = 61)		
	**N**	**%**	**N**	**%**	**N**	**%**	**N**	**%**	**N**	**%**	**P-value**
**Sex** ^*^											0.12
Female	183	78	45	75	49	83	47	86	42	69	
Male	52	22	15	25	10	17	8	14	19	31	
**Highest completed education level** ^*, †^											0.85
< 12 years^‡^	17	7	3	5	3	5	5	9	6	10	
12 years (high school diploma)	110	47	27	46	27	45	25	46	31	51	
University education	106	46	29	49	29	49	24	44	24	39	
**Health and lifestyle habits**											
Moderate physical activity, ≥ 3 times week^*^	163	69	38	63	40	68	39	71	46	75	0.53
Vigorous physical activity, ≥ 3 times/week^*^	66	28	9^a^	15	12^a^	20	13^a^	24	32^b^	53	**< 0.001**
Smoker^*,†^	40	17	11	19	12	20	9	17	8	13	0.74
Snuff user^*^	66	28	14	23	19	32	17	31	16	26	0.69
Vape user^*, †^	19	8	5	9	5	9	4	8	5	8	1.00
Intake of alcoholic beverages (≥ 1 times/week)^*^	129	55	29	48	37	63	33	60	30	49	0.27
**Daily use of iodized table salt** ^*^	184	78	41	68	48	81	43	78	52	85	0.13
**Habitual supplementation (≥ 1 times/week)** ^§^											
Multivitamin^*^	80	34	33^a^	55	23^a,b^	39	16^b,c^	29	8^c^	13	**< 0.001**
Vitamin D^*, ‖^	57	24	17	28	16	27	15	27	9	15	0.25
Vitamin B_12_^*, ‖^	41	17	20^a^	33	10	17	9	16	2^b^	3	**< 0.001**
Iron^*, ‖^	24	10	4	7	6	10	10	18	4	7	0.14
Vitamin C^*, ‖^	16	7	5	8	7	12	4	7	0	0	0.07
**Supplement use, 24-hour dietary recalls** ^§, ¶^											
Multivitamin^*^	67	29	32^a^	53	21^a, b^	36	11^b, c^	20	3^c^	5	**< 0.001**
Multivitamin containing vitamin A^*^	19	8	10^a^	17	4	7	4	7	1^b^	2	**0.027**
Multivitamin containing vitamin D^*^	43	18	23^a^	38	9^b^	15	8^b^	15	3^b^	5	**< 0.001**
Multivitamin containing B_12_^*^	67	29	32^a^	53	21^a, b^	36	11^b, c^	20	3^c^	5	**< 0.001**
Multivitamin containing calcium^*^	60	26	30^a^	50	19 ^a, b^	32	9^b, c^	16	2^c^	3	**< 0.001**
Multivitamin containing iron^*^	63	27	31^a^	52	21^a, b^	36	8^b, c^	15	3^c^	5	**< 0.001**
Multivitamin containing zinc^*^	64	27	31^a^	52	21^a, b^	36	9^b, c^	16	3^c^	5	**< 0.001**
Multivitamin containing selenium^*^	63	27	30^a^	50	20^a, b^	34	10^b, c^	18	3^c^	5	**< 0.001**
Multivitamin containing iodine^*^	61	26	28^a^	47	20^a, b^	34	10^b, c^	18	3^c^	5	**< 0.001**
Vitamin D^*, ‖^	31	13	13^a^	23	11^a^	19	5	9	2^b^	3	**0.01**
Vitamin B_12_^*, ‖^	21	9	15^a^	25	5	9	1^b^	2	0^b^	0	**< 0.001**
Iron^*, ‖^	18	8	6	10	5	9	5	9	2	3	0.51
**Season of participation**											0.12
Spring (Mar-May)^*^	79	34	19	32	16	27	22	40	22	36	
Summer (Jun-Aug)^*^	19	8	2	3	8	14	4	7	5	8	
Autumn (Sep-Nov)^*^	62	26	22	37	11	19	10	18	19	31	
Winter (Dec-Feb)^*^	75	32	17	28	24	41	19	35	15	25	
	**Mean**	**SD**	**Mean**	**SD**	**Mean**	**SD**	**Mean**	**SD**	**Mean**	**SD**	
**Age, years** ^**^	22	2	22	2	22	2	21	2	21	2	0.05
**BMI, kg/m** ^2**^	23	3	22	3	22	3	22	3	23	3	0.07

^*^Test for differences between dietary groups (categorical variables) using crosstabulations with Chi-square test or Fisher’s exact test, percentage (%) is shown for within group, and bolded p-values indicate significant differences between groups; ^†^Missing values for: education level (n = 2 reported ´other´), smoking (n = 4), and vape use (n = 3). ^‡^Of these, 16 were ≤ 19 years, whereof 13 were ≤ 18 years, and as such have not had the chance to complete high school. ^§^Other types of supplements (B-vitamin complex, folate, calcium, zinc, and magnesium) were reported by less than 5 per dietary group, and none reported iodine specific supplements; ^‖^Nutrient specific supplementation and not including multivitamins which contain the nutrient; ^¶^Shows the number and percentage of participants who reported the nutrient specific dietary supplement in at least one of the 24-hour dietary recalls;^**^Test for differences between dietary groups using one-way ANOVA with Bonferroni post hoc test. Unlike letters (^a, b, c^) within the same row indicate between which dietary groups there is a significant difference after correction for multiple analysis. Adjusted p-value of < 0.05 was accepted as statistically significant

There were no significant differences between the dietary groups for mean BMI (23 ± 3 kg/m^2^), mean age (22 ± 2 years), highest completed education level (47% had only a high school degree, and 46% had university education), or use of tobacco/nicotine products (17% smoked and 28% used snuff). However, omnivores (53%) reported a significantly higher frequency of vigorous physical activity (≥ 3 times/week) compared to all other dietary groups (vegans 15%, lacto-ovo-vegetarians 20%, pescatarians 24%). Most youth (67%) participated between October and March (autumn/winter). The majority of the youth eating plant-based diets had followed their dietary practice for 3–5 years (vegans 35%, lacto-ovo-vegetarians 34%, pescatarians 24%) or 5–9 years (vegans 35%, lacto-ovo-vegetarians 27%, pescatarians 47%), *p* = 0.06 (excluding omnivores).

## Micronutrient intake of youth eating plant-based or omnivorous diets

Usual intake of energy and micronutrients from total intakes (food and supplements combined), and foods only, is presented as median values with 25th and 75th percentile in Table 2. All four 24HDRs were completed by 99% of participants. Two participants completed three 24HDRs and one completed two 24HDRs.

**Table 2:** Usual daily intake of energy and micronutrients (median value with 25th and 75th percentile) from total intake (foods and supplements combined) and foods only in youth adhering to vegan, lacto-ovo-vegetarian, pescatarian or omnivorous diets (*n* = 235)

	Total sample (*n* = 235)		Vegan (*n* = 60)		Lacto-ovo -vegetarian (*n* = 59)		Pescatarian (*n* = 55)		Omnivore (*n* = 61)		
Usual daily intake^*^	Median	25th, 75th	Median	25th, 75th	Median	25th, 75th	Median	25th, 75th	Median	25th, 75th	P-value^†^
**Energy intake, MJ/d**	9	8, 10	9	8, 10	9^a^	8, 11	9^a^	8, 10	10^b^	9, 11	**0.016**
**Vitamin A (RE)**											
Total intake^‡^	708	508, 891	531^a^	425, 792	723^b^	540, 920	740^b^	620, 888	729^b^	569, 925	**0.003**
Foods only	691	491, 872	491^a^	416, 687	719^b^	508, 895	730^b^	620, 877	729^b^	569, 914	**< 0.001**
**Vitamin D (µg)**											
Total intake^‡^	7	4, 13	8	3, 22	5	3, 12	7	5, 9	7	5, 11	0.18
Foods only	5	3, 7	4^a^	2, 6	4^a^	3, 5	6^b^	5, 7	7^b^	5, 10	**< 0.001**
**Vitamin E (α-TE)**											
Total intake^‡^	19	16, 24	22^a^	18, 30	19^b^	15, 24	19^b^	16, 22	18^b^	15, 21	**< 0.001**
Foods only	19	16, 22	21^a^	18, 25	18	15, 22	18^b^	16, 20	17^b^	15, 20	**< 0.001**
**Vitamin K (µg)**											
Total intake^‡^	94	73, 118	97^a^	77, 132	99^a^	82, 118	97	61, 115	77^b^	61, 116	**0.01**
Foods only	90	71, 116	92	77, 120	95	82, 113	93	61, 115	77	61, 116	0.04^§^
**Vitamin B**_**1**_ **(mg/MJ)**											
Total intake^‡^	1	1, 2	2^a^	1, 3	1^b^	1, 1	1^b^	1, 1	1	1, 2	**< 0.001**
Foods only	1	1, 2	1^a^	1, 2	1	1, 1	1^b^	1, 1	1^a^	1, 2	**0.004**
**Vitamin B**_**2**_ **(mg)**											
Total intake^‡^	2	1, 2	2	1, 3	1	1, 2	2	1, 2	2	1, 2	0.05
Foods only	1	1, 2	1^a^	1, 1	1^b^	1, 2	1^c^	1, 2	2^d^	1, 2	**< 0.001**
**Vitamin C (mg)**											
Total intake^‡^	102	82, 137	121^a^	95, 149	106	81, 141	97^b^	80, 133	93^b^	77, 117	**0.005**
Foods only	96	77, 120	99	82, 131	102	74, 120	92	74, 113	92	75, 110	0.23
**Niacin (NE/MJ)**											
Total intake^‡^	28	23, 37	25^a^	21, 31	24^a^	21, 29	27^a^	22, 32	39^b^	32, 49	**< 0.001**
Foods only	27	22, 35	23^a^	20, 28	24^a^	21, 29	27^a^	22, 32	39^b^	32, 49	**< 0.001**
**Vitamin B**_**6**_ **(mg)**											
Total intake^‡^	2	2, 3	3^a^	2, 4	2^b^	1, 2	2^b^	1, 2	2	2, 3	**< 0.001**
Foods only	2	1, 2	2	2, 3	2^a^	1, 2	2^a^	1, 2	2^b^	2, 3	**< 0.001**
**Vitamin B**_**12**_ **(µg)**											
Total intake^‡^	4	3, 9	9^a^	3, 22	3^b^	2, 11	4^b^	3, 5	4	3, 6	**0.005**
Foods only	3	2, 4	1^a^	1, 2	2^b^	2, 3	3^c^	3, 5	4^c^	3, 6	**< 0.001**
**Folate (µg)**											
Total intake^‡^	376	313, 466	447^a^	368, 558	395^a, c^	320, 488	353^c^	296, 429	332^b, c^	292, 404	**< 0.001**
Foods only	363	308, 433	403^a^	346, 466	371^a, c^	320, 458	345^c^	291, 389	325^b, c^	288, 386	**< 0.001**
**Calcium (mg)**											
Total intake^‡^	935	717, 1140	730^a^	625, 991	989^b^	761, 1231	1020^b^	896, 1146	937^b^	716, 1187	**< 0.001**
Foods only	883	684, 1087	677^a^	544, 792	966^b^	731, 1140	1008^b^	896, 1128	890^b^	715, 1187	**< 0.001**
**Iron (mg)**											
Total intake^‡^	13	10, 19	19^a^	13, 24	14^b^	9, 20	11^b^	9, 14	12^b^	10, 14	**< 0.001**
Foods only	11	9, 14	13^a^	11, 16	11^b, c^	9, 13	10^b^	9, 11	12^c^	10, 14	**< 0.001**
**Zinc (mg)**											
Total intake^‡^	10	8, 15	12	8, 18	10	8, 17	9^a^	8, 11	12^b^	9, 14	**0.018**
Foods only	9	8, 11	8^a^	7, 9	9^a^	7, 11	9^a^	8, 10	12^b^	9, 14	**< 0.001**
**Magnesium (mg)**											
Total intake^‡^	391	324, 487	461^a^	368, 541	391^b^	331, 471	368^b^	292, 422	374^b^	299, 460	**< 0.001**
Foods only	383	321, 464	440^a^	359, 506	389	328, 439	362^b^	292, 398	366^b^	297, 440	**< 0.001**
**Phosphorous (mg)**											
Total intake^‡^	1317	1101, 1595	1257	1110, 1628	1264	1095, 1499	1383	1181, 1535	1421	1022, 1662	0.63
Foods only	1316	1099, 1597	1256	1110, 1628	1264	1095, 1499	1374	1181, 1535	1421	1022, 1662	0.63
**Potassium (mg)**											
Total intake^‡^	2967	2518, 3422	3030	2597, 3420	2944	2467, 3295	2785	2395, 3238	3102	2736, 3753	0.06
Foods only	2954	2506, 3423	3030	2568, 3419	2922	2422, 3277	2785^a^	2395, 3238	3102^b^	2736, 3753	**0.04**
**Selenium (µg)**											
Total intake^‡^	42	28, 59	37	25, 71	34^a^	25, 54	41	30, 56	49^b^	40, 59	**0.006**
Foods only	33	25, 46	26^a^	22, 31	28^a^	22, 34	37^b^	28, 47	48^b^	40, 57	**< 0.001**
**Iodine (µg)** ^¶^											
Total intake^‡^	160	125, 210	141	109, 240	152	115, 210	167	138, 210	170	131, 199	0.45
Foods only	142	115, 175	118^a^	95, 139	133^a^	110, 159	163^b^	135, 193	162^b^	131, 195	**< 0.001**

MJ/d, Megajoule per day

^*^Usual daily intakes were estimated by the Multiple Source Method using repeated 24-hour dietary recalls (between 2–4 days); ^†^For all variables, to test for differences between dietary groups, Kruskal-Wallis test with pairwise comparisons was used with Bonferroni post hoc test, and bolded p-values indicate a statistical significance between dietary groups; ^‡^Total intake entails foods and supplements combined; ^§^No difference between the dietary groups in the post hoc test; ^¶^Not including discretionary iodized household salt reported in the 24-hour dietary recalls; Unlike letters (^a, b,c, d^) in the same row indicate between which dietary groups there is a significant difference. Two-sided p-value of < 0.05 (adjusted) was accepted as statistically significant

### Usual intakes of energy and micronutrients from food and supplements

Omnivores (10 MJ/d) had a significantly higher energy intake compared to pescatarians (9 MJ/d) and lacto-ovo-vegetarians (9 MJ/d). From food and supplements combined, vegans had higher intakes of iron, vitamin E, and magnesium, and lower intakes of calcium and vitamin A compared to all other dietary groups, see Table 2. Moreover, vegans had higher intakes of vitamin B_1_, vitamin B_6_, and B_12_ compared to lacto-ovo-vegetarians and pescatarians, and higher intakes of vitamin C and folate compared to omnivores and pescatarians. Also, lacto-ovo-vegetarians had higher intakes of folate than omnivores. Furthermore, omnivores had higher intakes of niacin compared to all other dietary groups, higher intakes of zinc compared to pescatarians, higher intakes of selenium compared to lacto-ovo-vegetarians, and lower intakes of vitamin K compared to vegans and lacto-ovo-vegetarians. These differences were significant at the *p* < 0.05 level.

### Risk of inadequacy based on usual intakes from food and supplements

The percentage of participants with usual intakes of micronutrients (foods and supplements combined, and foods only) below the AR by NNR2023 is presented in Table 3.

**Table 3:** Proportion of youth adhering to vegan, lacto-ovo-vegetarian, pescatarian or omnivorous diets with usual daily intakes from total intake (foods and supplements combined) and foods only who do not meet the average requirements (AR) of micronutrients by the Nordic Nutrition Recommendations 2023 (*n* = 235)

			Total sample (*n* = 235)		Vegan (*n* = 60)		Lacto-ovo -vegetarian (*n* = 59)		Pescatarian (*n* = 55)		Omnivore (*n* = 61)		
	AR for females	AR for males	Below AR		Below AR		Below AR		Below AR		Below AR		
Usual daily intake^*^	18–24 yrs (15–17 yrs)	18–24 yrs (15–17 yrs)	N	%	N	%	**N**	%	**N**	%	**N**	%	P-value^†^
**Vitamin A (RE)**	540 (500)	630 (600)											
Total intake^‡^			94	40	34^a^	**57** ^§^	19^b^	32	14^b^	26	27	44	**0.003**
Foods only			113	48	44^a^	**73**	24^b^	41	16^b^	29	29^b^	48	**< 0.001**
**Vitamin D (µg)**	7.5 (7.5)	7.5 (7.5)											
Total intake^‡^			139	**59**	31	**52**	39	**66**	34	**62**	35	**57**	0.42
Foods only			191	**81**	57^a^	**95**	55^a, b^	**93**	42^b, c^	**76**	37^c^	**61**	**< 0.001**
**Vitamin E (α-TE)**	8 (9)^‖^	9 (10)^‖^											
Total intake^‡^			0	0	0	0	0	0	0	0	0	0	n/a
Foods only			0	0	0	0	0	0	0	0	0	0	n/a
**Vitamin K (µg)**	50 (45)^‖^	60 (50)^‖^											
Total intake^‡^			18	8	1^a^	2	0^a^	0	6	11	11^b^	18	**< 0.001**
Foods only			18	8	1^a^	2	0^a^	0	6	11	11^b^	18	**< 0.001**
**Vitamin B**_**1**_ **(mg/MJ)**	0.07 (0.07)	0.07 (0.07)											
Total intake^‡^			0	0	0	0	0	0	0	0	0	0	n/a
Foods only			0	0	0	0	0	0	0	0	0	0	n/a
**Vitamin B**_**2**_ **(mg)**	1.3 (1.3)	1.3 (1.3)											
Total intake^‡^			67	29	20	33	19	33	17	31	11	18	0.18
Foods only			93	40	39^a^	**65**	25^a, b^	42	18^b, c^	33	11^c^	18	**< 0.001**
**Vitamin C (mg)**	75 (75)	90 (85)											
Total intake^‡^			58	25	9	15	13	22	15	27	21	34	0.09
Foods only			69	30	15	25	16	27	16	29	22	36	0.57
**Niacin (NE/MJ)**	1.3 (1.3)	1.3 (1.3)											
Total intake^‡^			0	0	0	0	0	0	0	0	0	0	n/a
Foods only			0	0	0	0	0	0	0	0	0	0	n/a
**Vitamin B**_**6**_ **(mg)**	1.3 (1.3)	1.5 (1.5)											
Total intake^‡^			22	9	3^a^	5	15^b^	25	4	7	0^a^	0	**< 0.001**
Foods only			25	11	6	10	15^b^	25	4	7	0^a^	0	**< 0.001**
**Vitamin B**_**12**_ **(µg)**	3.2 (3.1)^‖^	3.2 (3.2)^‖^											
Total intake^‡^			76	32	15^a^	25	30^b^	**51**	16	29	11^a^	18	**< 0.001**
Foods only			140	**60**	57^a^	**95**	48^a^	**81**	24^b^	44	11^c^	18	**< 0.001**
**Folate (µg)**	250 (240)	250 (250)											
Total intake^‡^			14	6	1	2	2	3	3	6	8	13	0.07
Foods only			16	7	1^a^	2	2	3	3	6	10^b^	16	**0.006**
**Calcium (mg)**	870 (980)	870 (980)											
Total intake^‡^			104	44	41^a^	**68**	24^b, c^	41	10^c^	18	29^a, b^	48	**< 0.001**
Foods only			116	50	50^a^	**83**	26^b^	44	10^c^	18	30^b^	49	**< 0.001**
**Iron (mg)**	9 (9)	7 (9)											
Total intake^‡^			31	13	1^a^	2	9^b^	15	12^b^	22	9	15	**0.004**
Foods only			40	17	2^a^	3	13^b^	22	15^b^	27	10	16	**0.004**
**Zinc (mg)**	8.1 (10.2)	10.6 (11.7)											
Total intake^‡^			67	29	21^a^	35	24^a^	41	15	27	7^b^	12	**0.002**
Foods only			92	39	37^a^	**62**	28^a, b^	48	20^b^	36	7^c^	12	**< 0.001**
**Phosphorus (mg)**	440 (510)^‖^	440 (510)^‖^											
Total intake^‡^			0	0	0	0	0	0	0	0	0	0	n/a
Foods only			0	0	0	0	0	0	0	0	0	0	n/a
**Potassium (mg)**	2800 (2250)^‖^	2800 (2700)^‖^											
Total intake^‡^			99	42	25	42	24	41	29	**53**	21	34	0.26
Foods only			100	43	25	42	25	42	29	**53**	21	34	0.26
**Magnesium (mg)**	240 (200)^‖^	280 (240)^‖^											
Total intake^‡^			11	5	0^a^	0	2	3	2	4	7^b^	12	**0.02**
Foods only			11	5	0^a^	0	2	3	2	4	7^b^	12	**0.02**
**Selenium (µg)**	60 (55)^‖^	70 (70)^‖^											
Total intake^‡^			190	**81**	42	**70**	48	**81**	48	**87**	52	**85**	0.09
Foods only			224	**95**	60	**100**	57	**97**	52	**95**	55	**90**	0.08
**Iodine (µg)** ^¶^	120 (100)^‖^	120 (110)^‖^											
Total intake^‡^			54	23	20	33	16	27	7	13	11	18	0.04^††^
Foods only			73	31	32^a^	**53**	21	36	9^b^	16	11^b^	18	**< 0.001**

n/a, not applicable

^*^Usual daily intakes were estimated by the Multiple Source Method using repeated 24-hour dietary recalls (between 2–4 days); ^†^For all variables, to test for differences between dietary groups crosstabulations with Chi-square test or Fisher’s exact test was used, and the percentage (%) is shown within dietary groups; ^‡^Total intake entails foods and supplements combined; ^§^Bolded values in the table cells indicate which dietary groups demonstrate an increased risk for inadequate intake (> 50% of the group have intakes below the AR). ^‖^Provisional AR; ^¶^Excludes discretionary iodized household salt reported in the 24-hour dietary recalls; Bolded p-values indicate significant differences between dietary groups, and unlike letters (^a, b,c^) in the same row indicate between which groups the proportions differ in the post hoc test. ^††^No difference between the dietary groups in the post hoc test; Two-sided p-value of < 0.05 (adjusted) was accepted as statistically significant

From foods and supplements combined, vegans demonstrated a risk for inadequate intake of selenium (70% below AR), vitamin D (52% below AR), vitamin A (57% below AR) and calcium (68% below AR), while lacto-ovo-vegetarians demonstrated a risk for inadequate intake of selenium (81% below AR), vitamin D (66% below AR) and B_12_ (51% below AR). Also, pescatarians demonstrated a risk for inadequate intake from foods and supplements combined, of selenium (87% below AR), vitamin D (62% below AR), as well as of potassium (53% below AR), while omnivores demonstrated a risk for inadequate intake of selenium (85% below AR) and vitamin D (57% below AR) only. There were significant differences between the dietary groups in the proportion of youth with intakes, from foods and supplements combined, below the ARs of vitamins A, K, B_6_, B_12_, calcium, iron, zinc, and magnesium, see Table 3.

### Habitual supplementation and use of iodized salt

Vegans (55%) reported a higher habitual use (≥ 1 times/week in the past six months) of multivitamins compared to omnivores (13%) and pescatarians (29%), while lacto-ovo-vegetarians (39%) reported a higher habitual use of multivitamins compared to omnivores. Moreover, vegans reported the highest and omnivores the lowest habitual supplement use of B_12_ (33% vs. 3%). These differences were significant at the *p* < 0.01 level. Most participants (78%) reported daily use of an iodized table salt, and it did not significantly differ between the dietary groups.

## Biomarkers of nutritional status

The median (25th and 75th percentile) status of HB, sTfR, tHcy, MMA, 25-OH-D3, and UIC, and the proportion at risk of nutritional deficiencies is presented in Table 4.

**Table 4:** Biomarkers of nutritional status (iron, B_12_, vitamin D, and iodine) among youth adhering to vegan, lacto-ovo-vegetarian, pescatarian or omnivorous diets in sweden, and the proportion of youth at risk of nutritional deficiencies

	Total sample		Vegan		Lacto-ovo-vegetarian		Pescatarian		Omnivore		
Biomarkers of nutritional status	Median	25th, 75th	Median	25th, 75th	Median	25th, 75th	Median	25th, 75th	Median	25th, 75th	P-value
HB, g/L^*, †^	139	131, 149	140	133, 150	138	131, 148	138	129, 145	141	134, 150	0.65
sTfR, mg/L^*, †^	0.85	0.72, 0.98	0.88	0.75, 0.99	0.83	0.73, 0.97	0.84	0.65, 1.06	0.80	0.68, 0.95	0.37
tHcy, µmol/L^*, †^	4.0	2.9, 5.3	3.7	2.7, 5.3	4.4^a^	3.2, 5.5	4.5^a^	3.3, 5.6	3.4^b^	2.7, 4.4	**0.005**
MMA, µmol/L^*, †^	0.11	0.07, 0.16	0.13	0.07, 0.16	0.11	0.07, 0.18	0.10	0.07, 0.18	0.09	0.07, 0.13	0.82
25-OH-D3, nmol/L^*, †^	44	35, 55	39^a^	27, 52	42^a^	34, 52	53^b^	43, 58	44	35, 53	**< 0.001**
UIC, µg/L^†^	89	44, 165	80	39, 165	110	58, 178	95	46, 165	71	39, 140	0.22
	**N**	**%**	**N**	**%**	**N**	**%**	**N**	**%**	**N**	**%**	
**HB** ^*^											n/a
Anaemia, < 120 g/L (female), < 130 g/L (male)	0	0	0	0	0	0	0	0	0	0	
**sTfR** ^*^											n/a
Elevated status, > 3.09 mg/L^§^	0	0	0	0	0	0	0	0	0	0	
**tHcy** ^*, ‡^											0.57
Elevated status, > 15 µmol/L	2	1	1	2	1	2	0	0	0	0	
**MMA** ^*, ‡^											0.65
Depletion, > 0.27–0.37 µmol/L	10	5	3	6	3	6	2	5	2	4	
Risk for B_12_ deficiency, > 0.37 µmol/L	10	5	1	2	5	10	2	5	2	4	
**25-OH-D3** ^*, ‡^											**0.002**
Suboptimal status, 30–49 nmol/L	104	48	21	42	31	56	20	36	32	54	
Risk of deficiency, < 30 nmol/L	28	13	14^a^	28	6	11	4^b^	7	4^b^	7	
**UIC** ^‡^											0.30
≥ 100 µg/L^‖^	104	44	24	40	32	54	26	47	22	36	
50–99 µg/L^‖^	66	28	15	25	17	29	14	26	20	33	
20–49 µg/L^‖^	65	28	21	35	10	17	15	27	19	31	

HB, Haemoglobin. sTfR, Serum Transferrin Receptor. tHcy, Homocysteine. MMA, Methylmalonic acid. 25-OH-D3, 25-hydroxyvitamin D3. UIC, Urinary Iodine Concentration. n/a, not applicable

*Missing values: HB (*n* = 5), sTfR (*n* = 3), tHcy (*n* = 5), MMA (*n* = 45), 25-OH-D3 (*n* = 16); ^†^Test for differences between dietary groups using Kruskal-Wallis test with pairwise comparisons, and Bonferroni post hoc test; ^‡^To test for differences in proportions between dietary groups crosstabulations with Chi-square test or Fisher’s Exact test was used, and unlike letters (^a, b,c^) within the same row indicate which groups differ statistically; ^§^Elevated sTfR according to the assay and kit manual (EIA-5925 sTfR Human ELISA kit) was > 3.09 mg/L (normal range: 0.48–3.09 mg/L); ^‖^Percentage is provided for descriptive purposes - risk of deficiency is categorized on population level and not individual level; Blood biomarkers sTfR, tHcy, MMA and 25-OH-D3 were analysed from dried blood spots, HB from capillary blood using a HemoCue, and UIC from random spot urine samples. Biological samples were collected at different time points during the day and were non-fasted. For all tests, two-sided (adjusted) p-value of < 0.05 was accepted as statistically significant, and bolded p-values indicate significant differences between groups

### HB and sTfR as biomarkers of anaemia and iron insufficiency

Both HB and sTfR were within normal ranges across dietary groups, and there was no significant difference between the groups. None had HB values indicating a risk of anaemia (females: <120 g/L, males: <130 g/L) or elevated sTfR values indicative of iron insufficiency (> 3.09 mg/L).

### tHcy and MMA as functional biomarkers of B_12_ status

Omnivores had a significantly lower tHcy (3.4 µmol/L) compared to pescatarians (4.5 µmol/L) and lacto-ovo-vegetarians (4.4 µmol/L), although still within normal range. MMA was within normal range across dietary groups. Two participants had elevated tHcy (> 15 µmol/L) whereof one also had an elevated MMA value (for the other participant MMA could not be measured). Of the sample, 10% (*n* = 20) had elevated MMA values (> 0.27 µmol/L). Lacto-ovo-vegetarians demonstrated the highest proportion (16%) with elevated MMA values, but it was not significantly higher compared to the other dietary groups (Table 4).

### 25-OH-D3 as a biomarker of vitamin D status

Vegans (39 nmol/L) and lacto-ovo-vegetarians (42 nmol/L) had a significantly lower 25-OH-D3 concentration compared to pescatarians (53 nmol/L). All groups except pescatarians demonstrated suboptimal concentrations of 25-OH-D3 (30–49 nmol/L). Of the total sample, 13% (*n* = 28) were found to be at risk of vitamin D deficiency (< 30 nmol/L). Vegans had a significantly higher proportion (28%) at risk of deficiency compared to pescatarians (7%) and omnivores (7%). For all dietary groups higher 25-OH-D3 concentration was found in habitual compared to non-habitual supplement users of vitamin D **(supplementary material)**. There was no significant difference in 25-OH-D3 concentration when stratified by season of participation (total sample, and within dietary groups) **(supplementary material)**.

### UIC as a biomarker of iodine status

The median UIC (25th, 75th) for the total sample was 89 µg/L (44, 165) indicating a risk of inadequate iodine intake. Inadequate UIC (< 100 µg/L) was found in vegans at 80 µg/L (39, 165), pescatarians at 95 µg/L (46, 165) and omnivores at 71 µg/L (39, 140) (Table 4).

## Discussion

In this cross-sectional study of 16-to-24-year-olds in Sweden, youth adhering to vegan, lacto-ovo-vegetarian, pescatarian or omnivorous diets had intakes of micronutrients mostly aligned with recommendations. However, all dietary groups had usual intakes that demonstrated an increased risk of inadequate intakes (> 50% with intakes below the AR by NNR2023) of some micronutrients, whereof vitamin D and selenium (all groups), vitamin A and calcium (vegans), B_12_ (lacto-ovo-vegetarians), and potassium (pescatarians). Insufficient concentration of serum 25-OH-D3 was observed in all groups except pescatarians, which mostly aligns with the dietary intake data. Median blood biomarkers of iron insufficiency (HB and sTfR) and functional B_12_ (MMA and tHcy) were within normal ranges across dietary groups, supporting the dietary data indicating low risk for inadequate intakes from food and supplements. The median UIC indicated inadequate iodine intake in vegans, pescatarians, and omnivores, which contradicts their dietary intake data showing low risk for inadequate iodine intakes from foods and supplements.

### Intakes and status of vitamin D

Our findings illustrate that Swedish youth, regardless of plant-based or omnivorous diet, face a challenge in meeting the nutritional recommendations of vitamin D. However, vegans and lacto-ovo-vegetarians required supplementation to reach intakes at a similar level as intakes of pescatarians and omnivores. This aligns with previous findings in youth following different types of plant-based or omnivorous diets in Norway (*n* = 165, 16–24 years) [12]. Furthermore, systematic reviews have shown that regardless of dietary practice (vegan, vegetarian, pescatarian, omnivorous), and regardless if being adult (≥ 18 years) [3] or a child/adolescent (2–18 years) [4] intakes of vitamin D do not meet recommendations, albeit most of these studies assessed intake from foods only.

In this study, insufficient vitamin D status (< 50 nmol/L) was observed in vegans, lacto-ovo-vegetarians and omnivores, supporting that habitual intakes of vitamin D are inadequate. In the national food consumption survey from 2016, it was observed that 71–82% of Swedish 17–18 year olds had intakes below the AR (7.5 µg/d), and 43–62% had insufficient vitamin D status in 2016 [8], and our findings are consistent with this.

Since oily fish and fortified products (milk, yoghurt, margarine) are the main food sources of vitamin D in the Swedish diet [8, 19], the Swedish Food Agency recommend vitamin D supplementation for some risk groups, that may include vegans, vegetarians, and individuals with low intakes of vitamin D fortified foods or oily fish [43]. Adolescents (mean age of 15 years) in Sweden have a low consumption of fish, approximately 1.6 times/week [11]. We previously reported that more pescatarians than omnivores in this study met the recommended intakes of at least 200 g/week of oily fish [25]. This may explain why omnivores had lower 25-OH-D3 concentration than pescatarians, suggesting that their vitamin D status may benefit from higher intakes of oily fish. Supplementation is particularly important for risk groups between August-April due to the limited skin synthesis of vitamin D through UVB exposure [44]. In our study, less than a third of the youth reported specific supplementation of vitamin D, even though over two thirds partook during the autumn/winter months. Furthermore, only 18% of the youth consumed a multivitamin which contained vitamin D. Notably, we observed a higher 25-OH-D3 concentration among habitual (≥ 1 times/week in the past six months) vs. non habitual users of vitamin D supplements **(supplementary material)**. Our findings indicate that youth in Sweden require support and more specific food knowledge and skills to ensure that nutritional recommendations of vitamin D are met through foods. Furthermore, since vegans and vegetarians demonstrated very low dietary intakes of vitamin D from foods only, they may need specific knowledge about appropriate supplementation (dosage and frequency), and use of fortified foods, to secure habitual adequate intakes of vitamin D.

### Intakes and status of B_12_

In our study, blood biomarkers of functional B_12_ support the dietary intake data demonstrating that youth eating plant-based or omnivorous diets are at low risk of inadequate B_12_ intakes from foods and supplements. However, our findings suggest that lacto-ovo-vegetarians are at an increased risk of inadequate B_12_ intakes since 51% of the lacto-ovo-vegetarians had usual intakes below the AR of 3.2 µg/d. Of note, in the updated NNR from 2023, the AR (provisional) for B_12_ was increased by more than twofold compared to the AR (1.4 µg/d) by the NNR from 2012 [6]. Nevertheless, 16% of lacto-ovo-vegetarians demonstrated elevated MMA values, indicative of B_12_ depletion or risk of deficiency [39]. Similarly, a study of German children and adolescents also reported that lacto-ovo-vegetarians had the highest proportion at risk of B_12_ deficiency compared to supplemented vegans and omnivores [45]. Lacto-ovo-vegetarians are a heterogenous dietary group and there is large variability in intakes of animal-sources of B_12_ (eggs and dairy). If intakes of eggs, dairy, and fortified plant-based foods are low when consuming a lacto-ovo-vegetarian diet, B_12_ supplementation is required [16], though youth with limited nutrition knowledge may not be aware of this. It is well-established that B_12_ supplementation is required when eating a vegan diet due to negligible quantities of B_12_ in unfortified plant-based foods [2]. Most of the participating vegans reported supplementation of B_12_ (either included in a multivitamin or a specific B_12_ supplement), thus achieving adequate intakes of B_12_. This finding aligns with previous research among youth and adults in Norway [12, 46] and adults in Finland [47], indicating an awareness of supplement requirements among vegans. Nevertheless, since B_12_ deficiency develops over several years [37, 39], youth need to be equipped with appropriate knowledge on how to secure sufficient intakes of B_12_ when eating lacto-ovo-vegetarian or vegan diets.

### Intakes and status of iodine

The dietary intake data in our study showed low risk for inadequate iodine intakes from food and supplements, yet the median UIC (< 100 µg/L) demonstrated a risk of inadequate intake in all groups except lacto-ovo-vegetarians (110 µg/L). Single-spot UIC samples only reflect recent intake as 90% of iodine is excreted in the urine within 24–48 h in healthy non-pregnant persons [48]. When examining the iodine intake from only the first 24HDR, lacto-ovo-vegetarians had the highest median intake of iodine from foods and supplements combined (196 µg), followed by omnivores (154 µg), vegans (150 µg) and pescatarians (143 µg). This may explain the higher median UIC demonstrated in lacto-ovo-vegetarians. However, there is a high day-to-day variability in both intakes of iodine and UIC [48, 49]. Thus, accurately assessing the iodine nutrition of a group requires a large sample size; a previous study demonstrated that 400 participants are necessary when using random single-spot UIC samples [49]. Furthermore, the cut-offs currently recommended to use by WHO for assessment of iodine status in healthy adults (not pregnant or lactating) are extrapolated from studies in school-aged children (6–12 years) [42]. But it has been suggested that these cut-offs for median UIC are too high for assessment of iodine deficiency in adult populations [50]. Notably, the WHO plan to update their guiding report from 2007 for assessment of iodine status. Therefore, our findings from UIC indicating a potential risk of inadequate iodine intakes based on random spot-samples should be interpreted cautiously.

Past studies have demonstrated mild iodine deficiency, based on median UIC, in pescatarians and omnivores, with vegans and vegetarians often demonstrating moderate deficiency (< 50 µg/L) [12, 17, 47, 51]. These findings have been observed in countries with a universal salt iodization program (e.g., Switzerland (25 µg iodine per gram table salt [51]), Finland (25 µg/g [18, 47]), and Germany (20 µg/g [17, 52])), and in countries such as Norway where voluntary fortification of salt is permitted, but at present only at a level of 5 µg/g [12, 18, 21]), and in the UK where there is low use and availability of iodized salt [53]. In comparison, Sweden has a voluntary iodization of table salt, at a level of 50 µg/g, which likely contributes to the adequate iodine nutrition previously found in the general (non-pregnant or lactating) population [8, 18, 20, 21]. It has been estimated that iodized salt provides more than 50% of Swedish adults iodine intake [20], and other important dietary sources are saltwater fish, cow’s milk, dairy and eggs [18, 21]. There are however significant challenges in assessing intakes of iodine, including the difficulty in quantifying iodine from iodized table salt used in cooking or at the table, and variations in iodine content depending on factors such as if the food/meal is homemade or premade [21, 48]. Modern-day diets of young people in Western countries may be comprised to large extent of premade and processed foods [12, 25, 54, 55], which do not commonly include iodized salt. As such, dietary habits in current youth such as reduced intakes of animal-sourced foods, choosing other types of household salts (herb salt, sea salt, mineral salt) instead of iodized table salt, and eating more premade foods/meals and less home-cooked meals may increase the risk for inadequate iodine nutrition. To the best of our knowledge this is the first study to assess the iodine status among youth adhering to plant-based diets in Sweden. Since inadequate iodine intake over time can impair thyroid function [21, 48], which is crucial for growth, and neurological development, further evaluation of iodine nutrition in this population is warranted.

### Intakes of selenium

We found that the majority (81%) of the sample in this study had selenium intakes below the AR, indicating a risk of inadequate intakes across dietary groups. Our results are consistent with previous findings of inadequate selenium intakes in young vegans (*n* = 30) and female omnivores (*n* = 15) [24] in Sweden, and Norwegian youth following lacto-ovo-vegetarian (*n* = 19) or omnivorous (*n* = 71) diets [12]. In Scandinavia the soil is selenium-deplete, thus selenium is added to conventional animal-feed in Sweden [56]. Consequently, for individuals excluding animal-sources of selenium (meat, fish, eggs) [56] there is an increased risk for inadequate intakes of selenium. Notably, lower intakes of selenium from foods only have been found in vegans vs. omnivores in Sweden [24], Denmark [57], and Finland [47]. In comparison to our findings, the national food survey, *Riksmaten Adolescents 2016–17*, reported that less than a third (16–26%) of 17–18 year olds in Sweden (*n* = 1000) [8] had selenium intakes from foods only below the AR (30 µg/d for females and 35 µg/d for males). Since then, the AR (provisional) for selenium has been increased twofold in the updated NNR2023 [6], and this is the recommendation the dietary intakes are compared to in this study. This explains why we found a very high percentage not meeting the ARs. Yet, when comparing usual intakes of selenium, all dietary groups in this study had selenium intakes from foods and supplements combined (vegan 37 µg/d; lacto-ovo-vegetarian 34 µg/d; pescatarian 41 µg/d; omnivore 49 µg/d) in alignment with the median usual intakes (from foods only) observed on national level (38–52 µg/d in 17–18 year olds [8]). This indicates that with the updated dietary recommendations, youth have an intake of selenium that fails to meet the AR, regardless of dietary practice.

There is large variability in selenium content in food due to the variation in selenium soil content globally, which is impacted by factors associated with geographical location [56]. Hence, there are challenges in accurately assessing selenium intakes due to constraints associated with Food Composition Databases, such as lack of nutritional information for some foods, or outdated values. The Swedish Food Agency´s Food Composition Database had comprehensive nutritional values, based primarily on laboratory analyses. However, there are risks of incorrect estimates of selenium intakes (both under- and overestimation), for example if consumption of imported legumes, nuts, and cereals is high, intakes may be slightly underestimated. Additionally, for some food items in the database, the reported selenium content may be outdated and not reflect changes in selenium soil content. Future research should include biomarkers of selenium intake, such as selenoproteinP, to enhance the evaluation of adequate selenium intakes. To increase intakes of selenium, legumes (e.g., lentils), imported cereals, and high selenium content nuts/seeds (e.g., brazil nuts, sesame seeds) can be chosen.

### Intakes of vitamin A and calcium among vegans

Our results indicating that vegans had an increased risk for inadequate intakes of vitamin A are in contrast to previous findings since adults and children/adolescents following vegan diets in other studies in general meet the nutritional recommendations of vitamin A [3, 4, 12]. In vegan diets, carotenoid rich fruits and vegetables and fortified added fats are primary sources of vitamin A. However, in our study intakes of these food groups did not differ between the dietary groups [25], and the median intake of fruits, berries and vegetables of the total sample was 310 g/d, below the recommendation of 500–800 g/d [6]. Since lacto-ovo-vegetarians, pescatarians, and omnivores also consumed animal-sources of vitamin A, including dairy and eggs, this may explain why vegans had relatively low intakes of vitamin A. Inadequate intakes of vitamin A is uncommon in Sweden [7]. Nevertheless, increased intakes of fruits, berries and vegetables among youth would support them in meeting the nutritional recommendations of vitamin A and complying with the food-based dietary guidelines.

Moreover, 68% of vegans had calcium intakes below the AR, indicating an increased risk of inadequate intakes. This is congruent with previous findings among young Swedish vegans [24], as well as overall findings from systematic reviews among children/adolescents [58] and adults [5] eating vegan diets. These consistent findings raise concern about the bone health of young vegans [58] since peak bone mass is reached during early adulthood, and adequate intakes of calcium, in addition to adequate intakes of vitamin D, are needed to build and maintain skeletal mass [33, 59]. Dairy products including cheese are key dietary sources, contributing to almost 50% of the calcium intake among Swedish adolescents [11]. In Sweden most plant-based milks (79%) and yoghurt alternatives (73%) are fortified with calcium, commonly 120 mg per 100 g, while some vegan cheeses (28%), and few plant-based cream alternatives (9%) are calcium-fortified [13]. Fortification policies differ globally, including across the Nordic countries, which leads to large variations in the nutritional content of plant-based dairy alternatives. Consequently, replacing dairy products with plant-based alternatives may not ensure adequate intakes of calcium, although youth may not have sufficient nutrition knowledge to be aware of this. To increase intakes of calcium when adhering to a vegan diet, or vegetarian diet with limited dairy products, calcium fortified plant-based dairy alternatives, leafy greens, nuts and seeds, pulses, and calcium-set tofu should be consumed often.

## Strengths and limitations

The strengths of this study include the similarity of participant characteristics across the dietary groups, and the high compliance with the dietary assessment method in combination with biomarker data. Furthermore, the short-term dietary intake data from the 24HDRs was used to estimate usual daily intakes by the statistical MSM [31, 32]. This adjusts for intravariability in intake, providing a more robust reflection of micronutrient intake distributions, and the proportion at increased risk of inadequate intakes. Moreover, there was complete nutritional information for 2,300 food items from the Swedish Food Composition Database. However, the database lacked nutrient values for some new products, including plant-based meat analogs and dairy alternatives. To address this, we approximated their nutritional content using similar items from the database, or used nutrition data available for the product, or through standardized recipes. Such substitutions introduce some variation in estimates of micronutrient intakes; however, it enabled an approximation of intake in the absence of comprehensive and specific nutrient data. Moreover, although the eligibility criteria in this study only required participants to have adhered to their dietary pattern for at least six months prior, likely insufficient to affect nutritional status, most (67%) youth eating plant-based diets had adhered to their diet for 3–9 years. Yet, it could be speculated that participants who had adhered to their plant-based diet for a longer duration are better equipped with skills and knowledge necessary to compose well-balanced plant-based diets, and to use appropriate supplementation.

A limitation of our study is the recruitment methods (convenience and snowball sampling) employed, which resulted in a high percentage of female participants (78%), thus limiting the generalizability of our results. As we did not recruit a sufficient number of males, we could not perform sex-stratified analyses as intended in our sample size calculations. Nevertheless, our sample size recruited is sufficiently powered to detect meaningful differences in intakes of key nutrients (e.g., energy, vitamin D, B_12,_ iron) between the four dietary groups based on *a posteriori* sample size calculations [60]. With regards to evaluation of nutritional deficiencies based on biomarkers, there are some considerations for the interpretations of our findings. First, due to our sample size there is a risk for Type II errors. Second, sTfR and HB show values outside of the normal range when iron stores are depleted [36], therefore we cannot evaluate if iron stores were in a stage of depletion, but not yet depleted. Additionally, our findings which showed that no participants had elevated sTfR, indicative of iron insufficiency, should be interpreted cautiously given the lack of standardized assays and established cut-offs for sTfR. Third, evaluation of vitamin D deficiency is based on only serum 25-OH-D3 concentrations in this study, and not total 25-OH-D (25-OH-D3 + 25-OH-D2). Vitamin D2 is found naturally in some plant-based foods (e.g., mushrooms), and is the form of vitamin D included in some multivitamins or used to fortify plant-based dairy alternatives. Therefore, total 25-OH-D concentrations may be somewhat higher. As such, the proportion at risk for vitamin D deficiency in this study may be slightly overestimated [61]. Lastly, we collected only one random spot urine sample per participant. Although this method is appropriate and recommended by WHO for assessing population-level iodine status [42], a much larger sample is recommended [41]. Also, we did not control for factors such as collection time, hydration status and recent dietary intake which may have influenced our results [49]. Despite these limitations, our findings provide a preliminary indication of iodine status in current youth adhering to plant-based or omnivorous diets, but further research is warranted.

## Conclusion

In conclusion, this study demonstrates that youth adhering to plant-based or omnivorous diets achieve micronutrients intakes mostly aligned with recommendations through food and supplements. Yet, all dietary groups have nutritional shortcomings. Usual intakes of selenium and vitamin D (supported by 25-OH-D3 concentrations) demonstrated an increased risk of inadequacy across dietary groups, with additional risks for calcium and vitamin A in vegans, B_12_ (supported by MMA values) in lacto-ovo-vegetarians, and potassium in pescatarians. While usual intakes of iodine demonstrated low risk for inadequate intakes across dietary groups, median UIC suggested potential inadequacy in vegans, pescatarians, and omnivores. However, given the limited sample size, further research on iodine insufficiency is warranted. To prevent nutritional deficiencies, youth in Sweden require appropriate support on how to ensure adequate intakes of micronutrients, in particular vitamin D and selenium, and nutrients important for growth and development.

## Electronic supplementary material

Below is the link to the electronic supplementary material.

Supplementary Material 1

Supplementary Material 2

## Data Availability

The data generated and analyzed in the current study is not publicly available due to ongoing analysis. However, data can be made available on reasonable request.

## Declarations

### Standards of reporting

A STROBE checklist for reporting of observational studies has been provided with submission.
