# Supplementary material for: Micronutrient intake and nutritional status in 16-to-24-year-olds adhering to vegan, lacto-ovo-vegetarian, pescatarian or omnivorous diets in Sweden
Source: Eur J Nutr. 2025 Jun 26;64(5):231. doi: 10.1007/s00394-025-03738-2 (PMC12202508; doi:10.1007/s00394-025-03738-2)
Supplement: Supplementary file 1 — Supplementary Material 1 [file 394_2025_3738_MOESM1_ESM.pdf]

Supplementary material

Title: Micronutrient intake and nutritional status in 16-to-24-year-olds adhering to vegan, lacto-ovo-vegetarian, pescatarian or omnivorous diets in Sweden.

Authors: Isabelle Mulkerrins[1*], Anine Christine Medin[2], Synne Groufh-Jacobsen[2], Claire Margerison[3] and Christel Larsson[1].

[1] Department of Food and Nutrition, and Sport Science, Faculty of Education, University of Gothenburg, PO Box 300. 405 30 Gothenburg, Sweden.

[2] Department of Nutrition and Public Health, Faculty of Health and Sport Sciences, University of Agder, Universitetsveien 25, 4630 Kristiansand, Norway.

[3] Deakin University, Institute for Physical Activity and Nutrition, Melbourne, Australia.

***Corresponding author:** Department of Food and Nutrition, and Sport Science, Faculty of Education, University of Gothenburg, PO Box 300, SE-405 30 Gothenburg, Sweden.

E-mail: Isabelle.mulkerrins@gu.se

Supplementary Tables included:

Supplemental Table 1. Biomarkers of nutritional status of iron, B_{12} and vitamin D (median and 25th and 75th percentile) in Swedish youth, stratified by habitual vs. non-habitual supplement users and dietary practice.

Supplemental Table 2. Median 25-OH-D3 concentration in Swedish youth stratified by season of study participation and dietary practice.

Supplemental Table 1. Biomarkers of nutritional status of iron, B_{12} and vitamin D (median and 25th and 75th percentile) in Swedish youth, stratified by habitual vs. non-habitual supplement users and dietary practices.

Biomarker	**Vegan**				**Lacto-ovo-vegetarian**				**Pescatarian**				**Omnivore**			
	Nutrient specific supplement user[*]		*Non-supplement user*[†]		*Nutrient specific supplement user*[*]		*Non-supplement user*[†]		*Nutrient specific supplement user*[*]		*Non-supplement user*[†]		*Nutrient specific supplement user*[*]		*Non-supplement user*[†]	
	**50th (25th, 75th)** ‡	**N**	**50th (25th, 75th)** ‡	**N**	**50th (25th, 75th)** ‡	**N**	**50th (25th, 75th)** ‡	**N**	**50th (25th, 75th)** ‡	**N**	**50th (25th, 75th)** ‡	**N**	**50th (25th, 75th)** ‡	**N**	**50th (25th, 75th)** ‡	**N**
sTfR, mg/L[§]	0.86 (0.82, 0.87)	4	0.89 (0.74, 1.02)	56	0.89 (0.76, 1.06)	6	0.83 (0.73, 0.96)	52	0.9 (0.8, 1.2)	9	0.84 (0.65, 1.03)	45	0.88 (0.78, 1.41)	4	0.79 (0.67, 0.95)	56
HB, g/L[§]	135 (135, 146)	4	141 (132, 150)	54	143 (139, 153)	6	137 (131, 148)	53	137 (128, 152)	10	138 (129, 145)	43	134 (124, 141)	4	141 (135, 150)	56
MMA, µmol/L[¶]	0.13 (0.07, 0.15)	14	0.12 (0.07, 0.16)	34	0.06 (0.05, 0.24)	7	0.11 (0.07, 0.17)	41	0.16 (0.08, 0.24)	8	0.10 (0.07, 0.16)	36	0.09 (0.09, 0.09)	1	0.10 (0.07, 0.13)	49
tHcy, µmol/L[¶]	3.7 (2.9, 5.3)	18	3.7 (2.6, 5.3)	39	3.6 (2.9, 4.6)	10	4.7 (3.3, 5.6)	48	4.1 (3.2, 4.7)	9	4.61 (3.6, 5.7)	45	2.2 (1.8, 2.6)	2	3.5 (2.7, 4.4)	59
25-OH-D3, nmol/L[ǀ]	**51** (40, 54) ‡	17	**35** (25, 42) ‡	33	**51** (37, 59) ‡	16	**42** (32, 49) ‡	39	**66** (55, 76) ‡	15	**48** (39, 56) ‡	40	53 (45, 65)	7	42 (33, 53)	52
	Multivitamin user[*]		*Non-multivitamin user*[†]		*Multivitamin user*[*]		*Non-multivitamin user*[†]		*Multivitamin user*[*]		*Non-multivitamin user*[†]		*Multivitamin user*[*]		*Non-multivitamin user*[†]	
sTfR, mg/L	0.87 (0.76, 0.95)	33	0.93 (0.73, 1.19)	27	0.77 (0.71, 0.94)	22	0.88 (0.75, 1.01)	36	0.86 (0.69, 1.01)	16	0.84 (0.65, 1.15)	38	0.78 (0.73, 0.87)	8	0.80 (0.66, 0.97)	52
HB, g/L	136 (128, 144)	31	143 (133, 154)	27	140 (131, 151)	23	137 (130, 147)	36	143 (136, 152)	16	137 (128, 142)	37	140 (131, 153)	8	141 (134, 150)	52
MMA, µmol/L	0.11 (0.07, 0.16)	27	0.13 (0.11, 0.16)	21	0.11 (0.07, 0.28)	19	0.10 (0.06, 0.17)	29	0.10 (0.07, 0.17)	12	0.11 (0.07, 0.18)	32	0.07 (0.06, 0.20)	5	0.10 (0.07, 0.13)	45
tHcy, µmol/L	**3.4** (2.4, 4.5) ‡	32	**4.3** (3.1, 6.2) ‡	25	3.9 (3.2, 5.0)	22	4.7 (3.3, 5.6)	36	4.2 (3.5, 5.1)	16	4.7 (3.2, 5.8)	38	2.7 (2.2, 3.8)	8	3.5 (2.7, 4.4)	53
25-OH-D3, nmol/L	39 (32, 51)	29	36 (25, 52)	21	49 (41, 53)	22	39 (33, 49)	33	54 (41, 63)	16	52 (43, 57)	39	**53** (48, 71)	8	**42** (33, 53) ‡	51

sTfR= Serum Transferrin Receptor. HB= Haemoglobin. MMA = Methylmalonic Acid. tHcy = Homocysteine. 25-OH-D3 = 25-hydrxovyvitamin D3

*Supplement user is defined as having consumed the nutrient specific supplement once a week or more often in the past 6 months, and a multivitamin user was defined as having consumed a multivitamin once a week or more often the past six months; †Non-supplement user is defined as consuming the nutrient specific supplement less often than once a week or never in the past six months, and a non-multivitamin user was defined as having consumed a multivitamin less often than once a month or never the past six months; ‡Test of difference between supplement user vs. non-supplement users within each dietary group using Mann-Whitney U test, **bolded values** indicate statistically significant differences within the dietary group, $p<0.05$; §For sTfR and HB, supplement user was defined as having consumed a specific iron supplement, multivitamin was not included; ǀFor vitamin D, supplement user was defined as having consumed a specific vitamin D supplement, and multivitamin was not included; ¶For MMA and tHcy, supplement user was defined as having consumed a specific vitamin B_{12} supplement, and multivitamin was not included; Due to the small sample of supplement users in each dietary group, there is a risk for type II errors, therefore P-values are not shown, and data is provided for descriptive purpose.

Supplemental Table 2. Median 25-OH-D3 concentration in Swedish youth stratified by season of study participation and dietary practice.

	Two seasons					Four seasons							
	Summer (Jun-Nov)		Winter (Dec-May)			Spring (Mar-May)		Summer (Jun-Aug)		Autumn (Sep-Nov)		Winter (Dec-Feb)	
Dietary practice	**50th (25th, 75th)**	**N**	**50th (25th, 75th)**	**N**	**P-value***	**50th (25th, 75th)**	**N**	**50th (25th, 75th)**	**N**	**50th (25th, 75th)**	**N**	**50th (25th, 75th)**	**N**
**Total sample**	42 (33, 52)	77	46 (36, 56)	142	0.21	44 (33, 54)	45	46 (35, 53)	44	42 (33, 52)	44	50 (38, 57)	49
**Vegan**	42 (30, 52)	24	37 (26, 52)	36	0.42	37 (25,56)	19	57 (51, 62)	2	40 (29, 50)	22	37 (33, 42)	17
**Lacto-ovo-vegetarian**	41 (35, 46)	19	45 (34, 55)	40	0.29	42 (33, 50)	16	37 (33, 48)	8	41 (40, 44)	11	46 (34, 58)	24
**Pescatarian**	50 (42, 56)	14	53 (44, 59)	41	0.66	46 (38, 57)	22	46 (43, 51)	4	54 (42, 58)	10	56 (50, 66)	19
**Omnivore**	41 (33, 53)	24	45 (37, 55)	37	0.40	41 (33, 51)	22	47 (36, 53)	5	40 (32, 53)	19	50 (43, 58)	15

*To test for difference in 25-OH-D3 concentration within dietary groups stratified by season of participation, Mann- Whitney U test was used for two seasons (summer [June-November] and winter [December-May]). Due to a low number of participants during summer (June-August) the Kruskal-Wallis test of difference within diet groups is not sufficiently powered, and therefore p-values are not shown, and data is presented for descriptive purposes.
