# Supplementary material for: Micronutrient intake and nutritional status in 16-to-24-year-olds adhering to vegan, lacto-ovo-vegetarian, pescatarian or omnivorous diets in Sweden
Source: Eur J Nutr. 2025 Jun 26;64(5):231. doi: 10.1007/s00394-025-03738-2 (PMC12202508; doi:10.1007/s00394-025-03738-2)
Supplement: Supplementary file 2 — Supplementary Material 2 [file 394_2025_3738_MOESM2_ESM.pdf]

STROBE Statement—Checklist of items that should be included in reports of ***cross-sectional studies***

Title: Micronutrient intake and nutritional status among youth adhering to vegan, lacto-ovo-vegetarian, pescatarian or omnivorous diets in Sweden

	Item No	Recommendation	Page No
**Title and abstract**	1	(*a*) Indicate the study's design with a commonly used term in the title or the abstract	Pg 2. Abstract.
		(*b*) Provide in the abstract an informative and balanced summary of what was done and what was found	Pg 2.
**Introduction**			
Background/rationale	2	Explain the scientific background and rationale for the investigation being reported	Pg 3.
Objectives	3	State specific objectives, including any prespecified hypotheses	Pg 3.
**Methods**			
Study design	4	Present key elements of study design early in the paper	Pg 3.
Setting	5	Describe the setting, locations, and relevant dates, including periods of recruitment, exposure, follow-up, and data collection	Pg 3 and 4
Participants	6	(*a*) Give the eligibility criteria, and the sources and methods of selection of participants	Pg 3-4<br>A detailed description has been published previously
Variables	7	Clearly define all outcomes, exposures, predictors, potential confounders, and effect modifiers. Give diagnostic criteria, if applicable	Pg 4-7
Data sources/ measurement	8*	For each variable of interest, give sources of data and details of methods of assessment (measurement). Describe comparability of assessment methods if there is more than one group	Pg 4-7
Bias	9	Describe any efforts to address potential sources of bias	Pg 5. The use of the Multiple Source Method, which adjusts the short-term dietary intake data for inter- and intravariability of intakes and estimates usual daily intakes.<br><br>Pg 7. Subgroup analyses for nutritional status
Study size	10	Explain how the study size was arrived at	Pg 4.

Quantitative variables	11	Explain how quantitative variables were handled in the analyses. If applicable, describe which groupings were chosen and why	Pg 4, 5, 7
Statistical methods	12	(*a*) Describe all statistical methods, including those used to control for confounding	Pg 5 and 7
		(*b*) Describe any methods used to examine subgroups and interactions	Pg 7. Subgroup analyses for nutritional status
		(*c*) Explain how missing data were addressed	
		(*d*) If applicable, describe analytical methods taking account of sampling strategy	
		(*e*) Describe any sensitivity analyses	Pg 7. Subgroup analyses for nutritional status
**Results**			
Participants	13*	(a) Report numbers of individuals at each stage of study—eg numbers potentially eligible, examined for eligibility, confirmed eligible, included in the study, completing follow-up, and analysed	Pg 4.
		(b) Give reasons for non-participation at each stage	Pg 4.
		(c) Consider use of a flow diagram	
Descriptive data	14*	(a) Give characteristics of study participants (eg demographic, clinical, social) and information on exposures and potential confounders	Pg 8.
		(b) Indicate number of participants with missing data for each variable of interest	Pg 6 and 8.
Outcome data	15*	Report numbers of outcome events or summary measures	Pg 8-9 and tables.
Main results	16	(*a*) Give unadjusted estimates and, if applicable, confounder-adjusted estimates and their precision (eg, 95% confidence interval). Make clear which confounders were adjusted for and why they were included	Intakes, adjusted for inter-and intravariability in intakes<br>Pg 8-9
		(*b*) Report category boundaries when continuous variables were categorized	Pg 5-7 (method)<br>Pg 8-9 (results)
		(*c*) If relevant, consider translating estimates of relative risk into absolute risk for a meaningful time period	
Other analyses	17	Report other analyses done—eg analyses of subgroups and interactions, and sensitivity analyses	Pg 9. Vitamin D
**Discussion**			

Key results	18	Summarise key results with reference to study objectives	Pg 9-10
Limitations	19	Discuss limitations of the study, taking into account sources of potential bias or imprecision. Discuss both direction and magnitude of any potential bias	Pg 12.
Interpretation	20	Give a cautious overall interpretation of results considering objectives, limitations, multiplicity of analyses, results from similar studies, and other relevant evidence	Pg 12 and 13.
Generalisability	21	Discuss the generalisability (external validity) of the study results	Pg 12
**Other information**			
Funding	22	Give the source of funding and the role of the funders for the present study and, if applicable, for the original study on which the present article is based	Pg 14, Declaration statements

*Give information separately for exposed and unexposed groups.

Note: An Explanation and Elaboration article discusses each checklist item and gives methodological background and published examples of transparent reporting. The STROBE checklist is best used in conjunction with this article (freely available on the Web sites of PLoS Medicine at http://www.plosmedicine.org/, Annals of Internal Medicine at http://www.annals.org/, and Epidemiology at http://www.epidem.com/). Information on the STROBE Initiative is available at www.strobe-statement.org.
